# Supplementary material for: Antimicrobial resistance associations with national primary care antibiotic stewardship policy: Primary care-based, multilevel analytic study
Source: PLoS One. 2020 May 14;15(5):e0232903. doi: 10.1371/journal.pone.0232903 (PMC7224529; doi:10.1371/journal.pone.0232903)
Supplement: S1 Table — (DOCX) [file pone.0232903.s001.docx]

# **S1. Antibiotic dispensing data collected from NHS Digital**

| **Antibiotic Group** | **Antibiotic Name** |
| --- | --- |
| Cephalosporins | Cefalexin (1^st^) |
|  | Cefaclor (2^nd^) |
|  | Cefuroxime (2^nd^) |
| Macrolides | Azithromycin |
|  | Clarithromycin |
|  | Erythromycin |
| Penicillins | Amoxicillin |
|  | Co-amoxiclav |
|  | Flucloxacillin |
|  | Phenoxymethylpenicillin |
| Quinolones | Ciprofloxacin |
|  | Levofloxacin |
|  | Ofloxacin |
| Tetracyclines | Doxycycline |
|  | Lymecycline |
|  | Tetracycline |
| Trimethoprim | Trimethoprim |
| Others | Clindamycin |
|  | Metronidazole |
|  | Nitrofurantoin |
