# Supplementary material for: Antimicrobial resistance associations with national primary care antibiotic stewardship policy: Primary care-based, multilevel analytic study
Source: PLoS One. 2020 May 14;15(5):e0232903. doi: 10.1371/journal.pone.0232903 (PMC7224529; doi:10.1371/journal.pone.0232903)
Supplement: S2 Table — This procedure worked as follows: we trained the statistical model on 90% of the data (i.e. the training set), and then used this model to predict the remaining 10% of the data (i.e. the test set). The division between training and test set was made at random. A prediction accuracy, i.e. the percentage of cases in the test set for which the model predicted resistance correctly was then calculated. We repeated these two steps ten times, so that all observations had been part of the test set, and averaged the prediction accuracy over the ten test sets. This average prediction accuracy served as our criterion for statistical model performance. This procedure was repeated for all delays so that we could compare the statistical performance between the delays. (DOCX) [file pone.0232903.s002.docx]

# **S2. Cross-validation results comparing models with different time delays for antibiotic resistance**

|  |  | **Prediction Accuracy (in %)** | | |
| --- | --- | --- | --- | --- |
| **Dispensing** | **Resistance** | **Delay 1 Quarter** | **Delay 2 Quarter** | **Delay 3 Quarter** |
| Amoxicillin | Amoxicillin | 54.8 | 54.5 | 54.5 |
| Cefalexin | Cefalexin | 91.1 | 91.0 | 90.9 |
| Ciprofloxacin | Ciprofloxacin | 88.4 | 88.4 | 88.4 |
| Co-Amoxiclav | Co-Amoxiclav | 91.0 | 90.8 | 90.6 |
| Nitrofurantoin | Nitrofurantoin | 97.8 | 97.8 | 97.8 |
| Trimethoprim | Trimethoprim | 64.6 | 64.6 | 64.5 |
| All | Amoxicillin | 54.6 | 54.4 | 54.4 |
| All | Cephalexin | 91.1 | 91.0 | 90.9 |
| All | Ciprofloxacin | 88.4 | 88.4 | 88.4 |
| All | Co-Amoxiclav | 91.0 | 90.8 | 90.6 |
| All | Nitrofurantoin | 97.8 | 97.8 | 97.8 |
| All | Trimethoprim | 64.6 | 64.5 | 64.5 |
| Nitrofurantoin | Trimethoprim | 64.6 | 64.5 | 64.5 |

This procedure worked as follows: we trained the statistical model on 90% of the data (i.e. the training set), and then used this model to predict the remaining 10% of the data (i.e. the test set). The division between training and test set was made at random. A prediction accuracy, i.e. the percentage of cases in the test set for which the model predicted resistance correctly was then calculated. We repeated these two steps ten times, so that all observations had been part of the test set, and averaged the prediction accuracy over the ten test sets. This average prediction accuracy served as our criterion for statistical model performance. This procedure was repeated for all delays so that we could compare the statistical performance between the delays.
