# Supplementary material for: Antimicrobial resistance associations with national primary care antibiotic stewardship policy: Primary care-based, multilevel analytic study
Source: PLoS One. 2020 May 14;15(5):e0232903. doi: 10.1371/journal.pone.0232903 (PMC7224529; doi:10.1371/journal.pone.0232903)
Supplement: S3 Table — Numbers in bold indicate the five largest relative decreases in antibiotic dispensing (%) between 2013 and 2016 a % decrease in dispensed items between 2013 and 2016 b negative numbers indicate an increase in antibiotic items dispensed between 2013 and 2016. (DOCX) [file pone.0232903.s003.docx]

# **S3. Median number of dispensed antibiotic items/1000 registered practice population/year**

| **Antibiotic group** | **Antibiotic name** | **2013** | **2014** | **2015** | **2016** | **Relative Decrease, %^a^** |
| --- | --- | --- | --- | --- | --- | --- |
| **Primary care practices (N=163)** | | | | | | |
| Cephalosporins | Cefalexin (1^st^) | 34.3 | 36.7 | 32.9 | 27.6 | 19.53 |
|  | Cefaclor (2^nd^) | 0.7 | 0.7 | 0.5 | 0.4 | **42.86** |
|  | Cefuroxime (2^nd^) | 0.4 | 0.4 | 0.4 | 0.2 | **50.00** |
| Macrolides | Azithromycin | 10.2 | 11.9 | 13.5 | 15.2 | -32.89^b^ |
|  | Clarithromycin | 59.9 | 61.2 | 62.1 | 61.4 | -2.44^b^ |
|  | Clindamycin | 12.1 | 12.9 | 12.1 | 11.2 | 7.44 |
|  | Erythromycin | 53.4 | 46.6 | 37.0 | 30.4 | **43.07** |
| Penicillins | Amoxicillin | 274.8 | 255.7 | 243.4 | 237.5 | 13.57 |
|  | Co-amoxiclav | 107.6 | 99.1 | 75.6 | 55.2 | **48.70** |
|  | Flucloxacillin | 125.8 | 124.9 | 124.9 | 124.9 | 0.72 |
|  | Phenoxymethylpenicillin | 69.9 | 69.0 | 64.5 | 63.7 | 8.87 |
| Quinolones | Ciprofloxacin | 32.2 | 31.1 | 27.4 | 24.4 | **24.22** |
|  | Levofloxacin | 0.6 | 0.6 | 0.5 | 0.7 | -14.29^b^ |
|  | Ofloxacin | 2.9 | 2.8 | 2.7 | 2.9 | 0.00 |
| Tetracyclines | Doxycycline | 60.4 | 65.5 | 69.2 | 71.5 | -15.52^b^ |
|  | Lymecycline | 34.4 | 34.1 | 35.9 | 33.0 | 4.07 |
|  | Tetracycline | 0.8 | 0.7 | 0.7 | 0.8 | 0.00 |
| Trimethoprim | Trimethoprim | 133.5 | 131.8 | 128.2 | 122.2 | 8.46 |
| Others | Metronidazole | 32.6 | 31.4 | 30.4 | 29.7 | 8.90 |
|  | Nitrofurantoin | 82.7 | 82.6 | 92.5 | 88.5 | -6.55^b^ |

Numbers in bold indicate the five largest relative decreases in antibiotic dispensing (%) between 2013 and 2016

^a^ % decrease in dispensed items between 2013 and 2016

^b^ negative numbers indicate an increase in antibiotic items dispensed between 2013 and 2016
