# Supplementary material for: Antimicrobial resistance associations with national primary care antibiotic stewardship policy: Primary care-based, multilevel analytic study
Source: PLoS One. 2020 May 14;15(5):e0232903. doi: 10.1371/journal.pone.0232903 (PMC7224529; doi:10.1371/journal.pone.0232903)
Supplement: S4 Table — (DOCX) [file pone.0232903.s004.docx]

# **S4. Total number of primary care practice urine samples received between 2013 and 2016**

| **Year**  **(calendar)** | **Number of GP practices** | **Number of UTI specimens submitted** | **Number of unique patient *E. coli* UTI samples** |
| --- | --- | --- | --- |
| **2013** | 163 | 56,594 | 36,290 |
| **2014** | 163 | 60,021 | 39,316 |
| **2015** | 163 | 60,979 | 40,408 |
| **2016** | 163 | 52,297 | 36,689 |
| **Total (all years)** | **163** | **231,891** | **152,704** |
