# Supplementary material for: Antimicrobial resistance associations with national primary care antibiotic stewardship policy: Primary care-based, multilevel analytic study
Source: PLoS One. 2020 May 14;15(5):e0232903. doi: 10.1371/journal.pone.0232903 (PMC7224529; doi:10.1371/journal.pone.0232903)
Supplement: S5 Table — a data for amoxicillin resistance from Lab B only b data for cefalexin resistance from Lab A only for 2013 and 2014, then Lab A and Lab B from 2015 to 2016. (DOCX) [file pone.0232903.s005.docx]

# **S5. Number and percentage of resistant *E. coli* UTI per year**

| **Year** | **Amoxicillin^a^** | | **Cefalexin^b^** | | **Ciprofloxacin** | | **Co-amoxiclav** | | **Nitrofurantoin** | | **Trimethoprim** | |
| --- | --- | --- | --- | --- | --- | --- | --- | --- | --- | --- | --- | --- |
|  | **N tested** | **% Resistant** | **N**  **tested** | **% Resistant** | **N**  **tested** | **% Resistant** | **N**  **tested** | **% Resistant** | **N**  **tested** | **% Resistant** | **N**  **tested** | **% Resistant** |
| **2013** | 16,523 | 52.97 | 19,613 | 7.06 | 36,269 | 11.77 | 36,172 | 6.67 | 36,278 | 2.52 | 36,283 | 35.76 |
| **2014** | 18,035 | 53.29 | 21,264 | 8.11 | 40,911 | 11.84 | 39,283 | 8.08 | 39,651 | 2.14 | 39,308 | 35.73 |
| **2015** | 18,609 | 52.97 | 40,242 | 10.30 | 40,353 | 11.88 | 40,388 | 11.43 | 40,355 | 2.47 | 40,390 | 35.86 |
| **2016** | 18,411 | 50.54 | 36,494 | 8.82 | 36,648 | 10.76 | 36,636 | 9.41 | 36,656 | 2.06 | 36,670 | 35.37 |
| **Total** | 71,579 | 52.43 | 128,458 | 10.87 | 152,597 | 11.57 | 152,480 | 8.96 | 152,581 | 2.29 | 152,652 | 35.67 |

^a^ data for amoxicillin resistance from Lab B only

^b^ data for cefalexin resistance from Lab A only for 2013 and 2014, then Lab A and Lab B from 2015 to 2016.
