# Supplementary material for: Antimicrobial resistance associations with national primary care antibiotic stewardship policy: Primary care-based, multilevel analytic study
Source: PLoS One. 2020 May 14;15(5):e0232903. doi: 10.1371/journal.pone.0232903 (PMC7224529; doi:10.1371/journal.pone.0232903)
Supplement: S6 Table — Where ***p-value is <0.001; **p-value is <0.01; *p-value is <0.05; IMD = Index of Multiple Deprivation 2015; Urban = Urban/Rural Classification 2011 a The intercepts represent the average odds of observing resistance, keeping all else equal at the mean. i.e. an odds ratio of one indicates there is a 50% chance of observing resistance at the mean level of the covariates. (DOCX) [file pone.0232903.s006.docx]

# **S6. Relationship between rate of antibiotic dispensing and prevalence of antibiotic resistance within the same quarter (full table of results)**

|  | **Reduced dispensing of same antibiotic** | | **Reduced dispensing of all antibiotics** | | **Increased dispensing of nitrofurantoin** | |
| --- | --- | --- | --- | --- | --- | --- |
|  | **Odds ratio** | **95% CI** | **Odds ratio** | **95% CI** | **Odds ratio** | **95% CI** |
| **Amoxicillin resistance** |  |  |  |  |  |  |
| Intercept^a^ | 1.101** | 1.029 to 1.177 | 1.102** | 1.030 to 1.180 |  |  |
| Dispensing | 0.998* | 0.850 to 0.972 | 0.999** | 0.998 to 1.000 |  |  |
| Age | 0.993*** | 0.992 to 0.993 | 0.993*** | 0.992 to 0.993 |  |  |
| IMD 2015 | 0.996*** | 0.994 to 0.998 | 0.996*** | 0.994 to 0.998 |  |  |
| Urban | 1.010 | 0.947 to 1.079 | 1.010 | 0.946 to 1.079 |  |  |
| No. patients registered at GP practice | 1.000 | 1.000 to 1.000 | 1.000 | 1.000 to 1.000 |  |  |
| % of children under 5 years registered at GP practice | 0.969* | 0.943 to 0.995 | 0.963* | 0.937 to 0.991 |  |  |
| **Cefalexin resistance** |  |  |  |  |  |  |
| Intercept^a^ | 0.093*** | 0.086 to 0.100 | 0.090*** | 0.084 to 0.097 |  |  |
| Dispensing | 1.033*** | 1.020 to 1.046 | 1.001 | 1.000 to 1.002 |  |  |
| Age | 0.986*** | 0.985 to 0.987 | 0.986*** | 0.985 to 0.987 |  |  |
| IMD 2015 | 0.997* | 0.995 to 1.000 | 0.998* | 0.995 to 1.000 |  |  |
| Urban | 1.035 | 0.962 to 1.079 | 1.035 | 0.962 to 1.079 |  |  |
| No. patients registered at GP practice | 1.000 | 1.000 to 1.000 | 1.000 | 1.000 to 1.000 |  |  |
| % of children under 5 years registered at GP practice | 0.991 | 0.959 to 1.025 | 0.987 | 0.954 to 1.020 |  |  |
| **Ciprofloxacin resistance** |  |  |  |  |  |  |
| Intercept^a^ | 0.118*** | 0.109 to 0.128 | 0.118*** | 0.109 to 0.128 |  |  |
| Dispensing | 0.982* | 0.965 to 0.999 | 0.999* | 0.998 to 1.000 |  |  |
| Age | 0.979*** | 0.978 to 0.980 | 0.979*** | 0.978 to 0.980 |  |  |
| IMD 2015 | 0.996*** | 0.994 to 0.998 | 0.996*** | 0.994 to 0.998 |  |  |
| Urban | 1.107** | 1.038 to 1.181 | 1.107** | 1.038 to 1.181 |  |  |
| No. patients registered at GP practice | 1.000 | 1.000 to 1.000 | 1.000 | 1.000 to 1.000 |  |  |
| % of children under 5 years registered at GP practice | 0.973 | 0.934 to 1.016 | 0.976 | 0.934 to 1.018 |  |  |
| **Co-amoxiclav resistance** |  |  |  |  |  |  |
| Intercept^a^ | 0.087*** | 0.081 to 0.093 | 0.088*** | 0.082 to 0.094 |  |  |
| Dispensing | 1.014*** | 1.008 to 1.019 | 1.000 | 0.999 to 1.001 |  |  |
| Age | 0.985*** | 0.984 to 0.986 | 0.985*** | 0.984 to 0.986 |  |  |
| IMD 2015 | 0.994*** | 0.992 to 0.996 | 0.994*** | 0.992 to 0.996 |  |  |
| Urban | 1.025 | 0.959 to 1.094 | 1.027 | 0.962 to 1.096 |  |  |
| No. patients registered at GP practice | 0.989** | 0.981 to 1.000 | 1.000 | 1.000 to 1.000 |  |  |
| % of children under 5 years registered at GP practice | 0.958** | 0.931 to 0.985 | 0.963** | 0.937 to 0.989 |  |  |
| **Nitrofurantoin resistance** |  |  |  |  |  |  |
| Intercept^a^ | 0.016*** | 0.014 to 0.018 | 0.016*** | 0.014 to 0.018 |  |  |
| Dispensing | 1.012 | 0.997 to 1.027 | 0.998 | 0.996 to 1.000 |  |  |
| Age | 0.970*** | 0.968 to 0.972 | 0.970*** | 0.968 to 0.972 |  |  |
| IMD 2015 | 0.993*** | 0.988 to 0.997 | 0.993*** | 0.988 to 0.997 |  |  |
| Urban | 1.018 | 0.991 to 1.267 | 1.112 | 0.983 to 1.258 |  |  |
| No. patients registered at GP practice | 1.000 | 1.000 to 1.000 | 1.000 | 1.000 to 1.000 |  |  |
| % of children under 5 years registered at GP practice | 0.984 | 0.923 to 1.049 | 0.975 | 0.915 to 1.040 |  |  |
| **Trimethoprim resistance** |  |  |  |  |  |  |
| Intercept^a^ | 0.553*** | 0.527 to 0.581 | 0.554*** | 0.527 to 0.581 | 0.552*** | 0.527 to 0.579 |
| Dispensing | 0.996* | 0.992 to 1.000 | 0.999 | 0.999 to 1.000 | 0.994* | 0.989 to 0.999 |
| Age | 0.993*** | 0.992 to 0.994 | 0.993*** | 0.993 to 0.994 | 1.007*** | 1.006 to 1.007 |
| IMD 2015 | 0.998* | 0.997 to 1.000 | 0.998* | 0.997 to 1.000 | 1.002* | 1.000 to 1.003 |
| Urban | 1.015 | 0.973 to 1.058 | 1.016 | 0.974 to 1.060 | 0.983 | 0.943 to 1.025 |
| No. patients registered at GP practice | 1.000 | 1.000 to 1.000 | 1.000 | 1.000 to 1.000 | 1.000 | 1.000 to 1.000 |
| % of children under 5 years registered at GP practice | 1.001 | 0.978 to 1.025 | 1.004 | 0.980 to 1.028 | 0.993 | 0.971 to 1.016 |

Where ***p-value is <0.001; **p-value is <0.01; *p-value is <0.05; IMD = Index of Multiple Deprivation 2015; Urban = Urban/Rural Classification 2011

^a^ The intercepts represent the average odds of observing resistance, keeping all else equal at the mean. i.e. an odds ratio of one indicates there is a 50% chance of observing resistance at the mean level of the covariates.
