# Supplementary material for: Antimicrobial resistance associations with national primary care antibiotic stewardship policy: Primary care-based, multilevel analytic study
Source: PLoS One. 2020 May 14;15(5):e0232903. doi: 10.1371/journal.pone.0232903 (PMC7224529; doi:10.1371/journal.pone.0232903)
Supplement: S7 Table — Where ***p-value is <0.001; **p-value is <0.01; *p-value is <0.05; IMD = Index of Multiple Deprivation 2015; Urban = Urban/Rural Classification 2011 a The intercepts represent the average odds of observing resistance, keeping all else equal at the mean. i.e. an odds ratio of one indicates there is a 50% chance of observing resistance at the mean level of the covariates. (DOCX) [file pone.0232903.s007.docx]

# **S7. Relationship between rate of antibiotic dispensing and prevalence of antibiotic resistance in the subsequent quarter (full table of results)**

|  | **Reduced dispensing of same antibiotic** | | **Reduced dispensing of all antibiotics** | | **Increased dispensing of nitrofurantoin** | |
| --- | --- | --- | --- | --- | --- | --- |
|  | **Odds ratio** | **95% CI** | **Odds ratio** | **95% CI** | **Odds ratio** | **95% CI** |
| **Amoxicillin resistance** |  |  |  |  |  |  |
| Intercept^a^ | 1.088* | 1.015 to 1.165 | 1.085* | 1.012 to 1.163 |  |  |
| Dispensing | 0.997** | 0.995 to 0.999 | 0.999** | 0.998 to 1.000 |  |  |
| Age | 0.993*** | 0.992 to 0.993 | 0.993*** | 0.992 to 0.993 |  |  |
| IMD 2015 | 0.996*** | 0.994 to 0.998 | 0.996*** | 0.994 to 0.998 |  |  |
| Urban | 0.997 | 0.932 to 1.066 | 0.995 | 0.931 to 1.065 |  |  |
| No. patients registered at GP practice | 1.000 | 1.000 to 1.000 | 1.000 | 1.000 to 1.000 |  |  |
| % of children under 5 years registered at GP practice | 0.968* | 0.942 to 0.996 | 0.963* | 0.935 to 0.991 |  |  |
| **Cefalexin resistance** |  |  |  |  |  |  |
| Intercept^a^ | 0.093*** | 0.086 to 0.100 | 0.091*** | 0.084 to 0.098 |  |  |
| Dispensing | 1.033*** | 1.011 to 1.036 | 1.001 | 1.000 to 1.002 |  |  |
| Age | 0.987*** | 0.986 to 0.988 | 0.987*** | 0.986 to 0.988 |  |  |
| IMD 2015 | 0.997* | 0.995 to 1.000 | 0.997* | 0.995 to 1.000 |  |  |
| Urban | 1.034 | 0.960 to 1.115 | 1.028 | 0.954 to 1.107 |  |  |
| No. patients registered at GP practice | 1.000 | 1.000 to 1.000 | 1.000 | 1.000 to 1.000 |  |  |
| % of children under 5 years registered at GP practice | 0.991 | 0.959 to 1.026 | 0.988 | 0.955 to 1.022 |  |  |
| **Ciprofloxacin resistance** |  |  |  |  |  |  |
| Intercept^a^ | 0.115*** | 0.106 to 0.125 | 0.115*** | 0.106 to 0.125 |  |  |
| Dispensing | 0.982 | 0.965 to 1.000 | 1.000 | 0.998 to 1.001 |  |  |
| Age | 0.979*** | 0.978 to 0.980 | 0.979*** | 0.978 to 0.980 |  |  |
| IMD 2015 | 0.995*** | 0.993 to 0.997 | 0.995*** | 0.993 to 0.997 |  |  |
| Urban | 1.088* | 1.019 to 1.163 | 1.089* | 1.019 to 1.164 |  |  |
| No. patients registered at GP practice | 1.000 | 1.000 to 1.000 | 1.000 | 1.000 to 1.000 |  |  |
| % of children under 5 years registered at GP practice | 0.976 | 0.935 to 1.018 | 0.977 | 0.935 to 1.020 |  |  |
| **Co-amoxiclav resistance** |  |  |  |  |  |  |
| Intercept^a^ | 0.089*** | 0.083 to 0.095 | 0.089*** | 0.083 to 0.095 |  |  |
| Dispensing | 1.010*** | 1.004 to 1.016 | 1.000 | 0.999 to 1.001 |  |  |
| Age | 0.985*** | 0.984 to 0.986 | 0.985*** | 0.984 to 0.986 |  |  |
| IMD 2015 | 0.994*** | 0.992 to 0.996 | 0.994*** | 0.992 to 0.996 |  |  |
| Urban | 1.017 | 0.951 to 1.088 | 1.019 | 0.953 to 1.091 |  |  |
| No. patients registered at GP practice | 0.988** | 0.980 to 0.997 | 1.000 | 1.000 to 1.000 |  |  |
| % of children under 5 years registered at GP practice | 0.959** | 0.932 to 0.987 | 0.963** | 0.936 to 0.990 |  |  |
| **Nitrofurantoin resistance** |  |  |  |  |  |  |
| Intercept^a^ | 0.015*** | 0.013 to 0.017 | 0.015*** | 0.013 to 0.017 |  |  |
| Dispensing | 0.999 | 0.983 to 1.013 | 0.996*** | 0.994 to 0.998 |  |  |
| Age | 0.970*** | 0.968 to 0.972 | 0.970*** | 0.968 to 0.972 |  |  |
| IMD 2015 | 0.992** | 0.988 to 0.997 | 0.993** | 0.988 to 0.997 |  |  |
| Urban | 1.075 | 0.946 to 1.222 | 1.066 | 0.937 to 1.212 |  |  |
| No. patients registered at GP practice | 1.000 | 1.000 to 1.000 | 1.000 | 1.000 to 1.000 |  |  |
| % of children under 5 years registered at GP practice | 0.971 | 0.909 to 1.037 | 0.971 | 0.908 to 1.038 |  |  |
| **Trimethoprim resistance** |  |  |  |  |  |  |
| Intercept^a^ | 0.544*** | 0.518 to 0.572 | 0.544*** | 0.518 to 0.572 | 0.543*** | 0.518 to 0.570 |
| Dispensing | 0.992*** | 0.988 to 0.997 | 0.999* | 0.999 to 1.000 | 0.991*** | 0.986 to 0.996 |
| Age | 0.993*** | 0.993 to 0.994 | 0.993*** | 0.993 to 0.994 | 1.007*** | 1.006 to 1.007 |
| IMD 2015 | 0.998* | 0.997 to 1.000 | 0.998* | 0.997 to 1.000 | 1.002* | 1.000 to 1.003 |
| Urban | 0.999 | 0.957 to 1.043 | 1.002 | 0.960 to 1.046 | 0.996 | 0.954 to 1.040 |
| No. patients registered at GP practice | 1.000 | 1.000 to 1.000 | 1.000 | 1.000 to 1.000 | 1.000 | 1.000 to 1.000 |
| % of children under 5 years registered at GP practice | 1.000 | 0.977 to 1.024 | 1.004 | 0.980 to 1.029 | 0.991 | 0.969 to 1.014 |

Where ***p-value is <0.001; **p-value is <0.01; *p-value is <0.05; IMD = Index of Multiple Deprivation 2015; Urban = Urban/Rural Classification 2011

^a^ The intercepts represent the average odds of observing resistance, keeping all else equal at the mean. i.e. an odds ratio of one indicates there is a 50% chance of observing resistance at the mean level of the covariates.
